# Photocatalytic, Antimicrobial and Biocompatibility Features of Cotton Knit Coated with Fe-N-Doped Titanium Dioxide Nanoparticles

**DOI:** 10.3390/ma9090789

**Published:** 2016-09-21

**Authors:** Miruna Silvia Stan, Ionela Cristina Nica, Anca Dinischiotu, Elena Varzaru, Ovidiu George Iordache, Iuliana Dumitrescu, Marcela Popa, Mariana Carmen Chifiriuc, Gratiela G. Pircalabioru, Veronica Lazar, Eugenia Bezirtzoglou, Marcel Feder, Lucian Diamandescu

**Affiliations:** 1 Department of Biochemistry and Molecular Biology, Faculty of Biology, University of Bucharest, 91-95 Splaiul Independentei, 050095 Bucharest, Romania; miruna.stan@bio.unibuc.ro (M.S.S.); cristinai.nica@gmail.com (I.C.N.); 2 National R&D Institute for Textiles and Leather Bucharest (INCDTP), 16 Lucretiu Patrascanu, 030508 Bucharest, Romania; elena.varzaru@certex.ro (E.V.); iordacheovidiu.g@certex.ro (O.G.I.); 3 Department of Botanic-Microbiology, Faculty of Biology, University of Bucharest, 1-3 Aleea Portocalelor, 60101 Bucharest, Romania; bmarcelica@yahoo.com (M.P.); carmen.chifiriuc@gmail.com (M.C.C.); gratiela87@gmail.com (G.G.P.); lazar@botanic.unibuc.ro (V.L.); 4 Research Institute of the University of Bucharest—ICUB, University of Bucharest, 91-95 Splaiul Independentei, 050095 Bucharest, Romania; 5 Department of Agricultural Development, Democritus University of Thrace, 67100 Xanthi, Greece; empezirt@agro.duth.gr; 6 National Institute of Materials Physics (NIMP), Atomistilor 405A, 077125 Bucharest-Magurele, Romania; mfeder@infim.ro (M.F.); diamand@infim.ro (L.D.)

**Keywords:** TiO_2_-1% Fe-N, photocatalysis, textiles, antimicrobial properties, biocompatibility

## Abstract

Our research was focused on the evaluation of the photocatalytic and antimicrobial properties, as well as biocompatibility of cotton fabrics coated with fresh and reused dispersions of nanoscaled TiO_2_-1% Fe-N particles prepared by the hydrothermal method and post-annealed at 400 °C. The powders were characterized by X-ray diffraction (XRD), Mössbauer spectroscopy and X-ray photoelectron spectroscopy. The textiles coated with doped TiO_2_ were characterized by scanning electron microscopy and energy dispersive X-ray analyses, and their photocatalytic effect by trichromatic coordinates of the materials stained with methylene blue and coffee and exposed to UV, visible and solar light. The resulting doped TiO_2_ consists of a mixture of prevailing anatase phase and a small amount (~15%–20%) of brookite, containing Fe^3+^ and nitrogen. By reusing dispersions of TiO_2_-1% Fe-N, high amounts of photocatalysts were deposited on the fabrics, and the photocatalytic activity was improved, especially under visible light. The treated fabrics exhibited specific antimicrobial features, which were dependent on their composition, microbial strain and incubation time. The in vitro biocompatibility evaluation on CCD-1070Sk dermal fibroblasts confirmed the absence of cytotoxicity after short-term exposure. These results highlight the potential of TiO_2_-1% Fe-N nanoparticles for further use in the development of innovative self-cleaning and antimicrobial photocatalytic cotton textiles. However, further studies are required in order to assess the long-term skin exposure effects and the possible particle release due to wearing.

## 1. Introduction

The photodegradation of different environmental pollutants by photocatalysts, such as titanium dioxide (TiO_2_), is one of the most studied processes. Between many advantages, such as mineralization of toxic and bio-resistant organic compounds [1,2,3], and the inactivation of pathogenic microorganisms [4], one of the main limitations of using TiO_2_ is the high quantity needed to obtain the desired effect.

Consequently, high amounts of waste are generated and released into the aquatic environment via effluents, where nanoparticles may pose an ecological risk [5,6], while the photocatalyst recovery from the used dispersion is a very expensive process at the industrial scale [7]. One solution could be the multiple usage of the photocatalyst dispersion [8], even if the photo-catalytic activity decreases gradually [9,10], due to the presence of adsorbed contaminants on its surface [11], or to the nanoparticles’ agglomeration. Although many studies have been dedicated to the recovery or separation of TiO_2_ used for the treatment of contaminated waters [12,13,14] or to the regeneration of TiO_2_ immobilized on supports in the form of films [15], none of them were focused on the re-use of the TiO_2_-1% Fe-N dispersion resulting from the textiles’ treatment. By this approach, one could expect to reduce the amount of doped TiO_2_ released into the environment and, also, by using the same treatment bath, to decrease the consumption of water and chemicals.

It has been shown that the process of TiO_2_ doping with various metal or non-metal elements can extend or enhance their photoactivity under visible light, making it useful not only for the treatment of polluted water [16], but also for the development of antimicrobial agents and materials [17]. 

The nano TiO_2_-coated fabrics, as well as those impregnated with doped TiO_2_, can harbor significant antibacterial (against *Escherichia coli* and *Staphylococcus aureus*) and antifungal (against *Candida albicans*) [18] activity, as well as self-cleaning properties, while having no adverse effects on human dermal fibroblasts [19,20]. 

In the current work, cotton fabrics were treated with TiO_2_-1% Fe-N particles and with the dispersion remaining after the first treatment. The fabrics treated as described were characterized by scanning electron microscopy (SEM) and energy dispersive X-ray (EDX) analyses, while the modification of color parameters after staining with methylene blue (MB) and coffee was investigated after exposure to ultraviolet (UV) and visible light. In addition, the antimicrobial activity was evaluated against Gram-positive (*S. aureus*, *Enterococcus faecalis*, *Bacillus subtilis*) and Gram-negative (*Pseudomonas aeruginosa*, *E. coli*) bacterial reference strains using standardized quantitative assays of viable cell counts. Finally, biocompatibility was assessed by analyzing the cellular morphology, viability and cell membrane integrity of CCD-1070Sk normal human skin fibroblasts after 4 h of contact with the modified textiles in order to provide useful experimental data for further in vivo studies.

## 2. Results

### 2.1. Photocatalyst Characterization

#### 2.1.1. X-ray Diffraction Analysis

**Figure 1:**
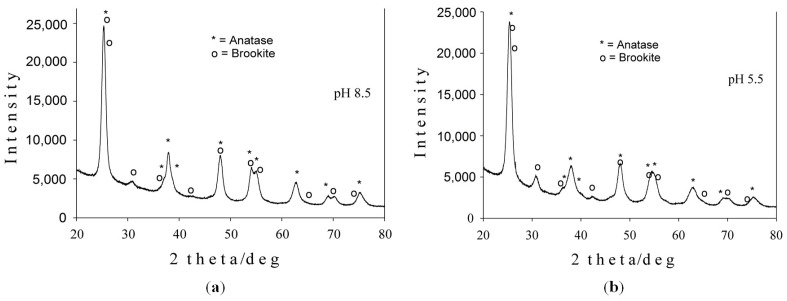
X-ray diffraction patterns of TiO_2_-1% Fe-N samples obtained at pH 8.5 (**a**) and pH 5.5 (**b**).

The X-ray diffractograms of hydrothermally-synthesized Samples 1 and 2 are shown in Figure 1. In both cases, the XRD patterns consisted of two phases: a prevailing tetragonal anatase phase (ICSD #9855) accompanied by the orthorhombic brookite (ICSD #15409) phase. The Rietveld refinement results for both samples are presented in Table 1. The brookite amount obtained at pH 5.5 was a little bit higher than at pH 8.5. The crystallite size given by the Scherrer formula [21] belongs to the range of 8–12 nm.

#### 2.1.2. Mössbauer Spectroscopy

**Figure 2:**
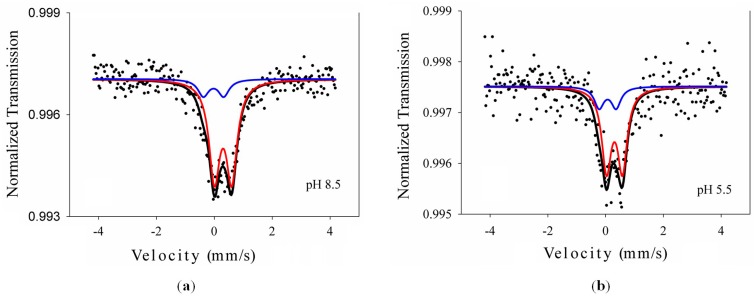
Mössbauer spectra of the hydrothermal samples at pH 8.5 (**a**) and 5.5 (**b**) together with the computer fit (continuous lines).

The presence of iron in the obtained samples was evidenced by ^57^Fe Mössbauer measurements at room temperature. The spectra (Figure 2) consisted of a central quadrupole pattern that was best deconvoluted into two doublets with Lorentzian lines. In good agreement with the XRD data, the most intense doublet corresponds to Fe^3+^ in the anatase phase (continuous red lines in Figure 2) and the less intense one to the brookite phase (blue lines in Figure 2). The characteristic Mössbauer hyperfine parameters isomer shift (IS), quadrupole splitting (ΔE_Q_), line width (Γ), together with the relative areas, as resulted from the fit with Lorentzian lines, are included in Table 2. The obtained parameters are consistent with the presence of Fe^3+^ in both anatase and brookite structures.

The presence of nitrogen in the studied samples was evidenced by X-ray photoelectron spectroscopy (XPS) in our previous study [22]. A determination of the total nitrogen ratio detected by XPS related to the total TiO_2_ revealed the presence of 0.6 at% N in the studied samples.

#### 2.1.3. Photocatalytic Test

**Figure 3:**
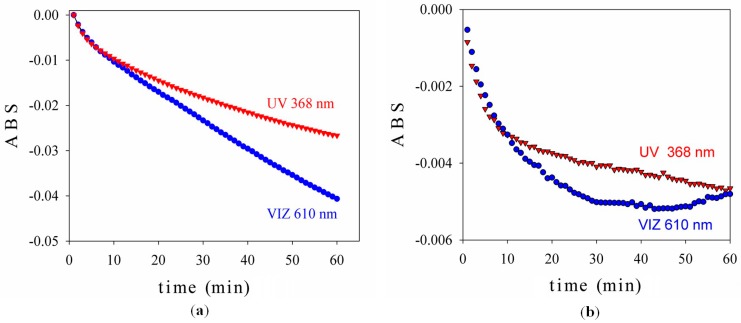
Photocatalytic degradation versus time of Sample 1 (**a**) and Sample 2 (**b**).

The nanoscaled TiO_2_-1% Fe-N samples prepared under hydrothermal conditions followed by post-annealing at 400 °C/2 h were used to prepare thin films on quartz support. In a suspension containing the obtained nanopowders, polyethylene glycol (PEG 600) was added in order to ensure the best adherence of nanoparticles to the quartz surface. Before the photocatalytic test, the samples were calcined to remove PEG, then cleaned by exposure under a 30-W UV lamp (λ = 365 nm) for 2 h. After cleaning, the films were immersed for 2 h in methylene blue and finally dried at room temperature. The photocatalytic activity was measured by the intensity of pulsed light (visible 610 nm) reflected from the sample surfaces coated with MB, over a period of 60 min. In Figure 3, the absorbance (ABS), as given by the PCC-2 (ULVAC, Chigasaki, Kanagawa, Japan) tester in both visible and UV radiation, is shown for Samples 1 and 2. ABS is a measure of the MB degradation in time, as a result of the photocatalytic activity of TiO_2_-1% Fe-N particles, under UV or visible light irradiation during measurements. The decomposition of MB under UV or visible light irradiation leads to a gradual decrease of optical absorption; thus, a higher negative ABS value means a better photocatalytic activity. A better activity in the visible spectrum in comparison with the UV light activity was noticed in the case of Sample 1 (pH = 8.5). In contrast, the photocatalytic activity of Sample 2 was much lower than of Sample 1 in both the UV and visible regions, with a clear saturation tendency. The most probable key elements to explain the differences are the brookite content, which is higher in Sample 2, accompanied by surface peculiarities, absorbance properties and band gap energies.

### 2.2. Characterization of Treated Textile Materials by SEM-EDX

**Figure 4:**
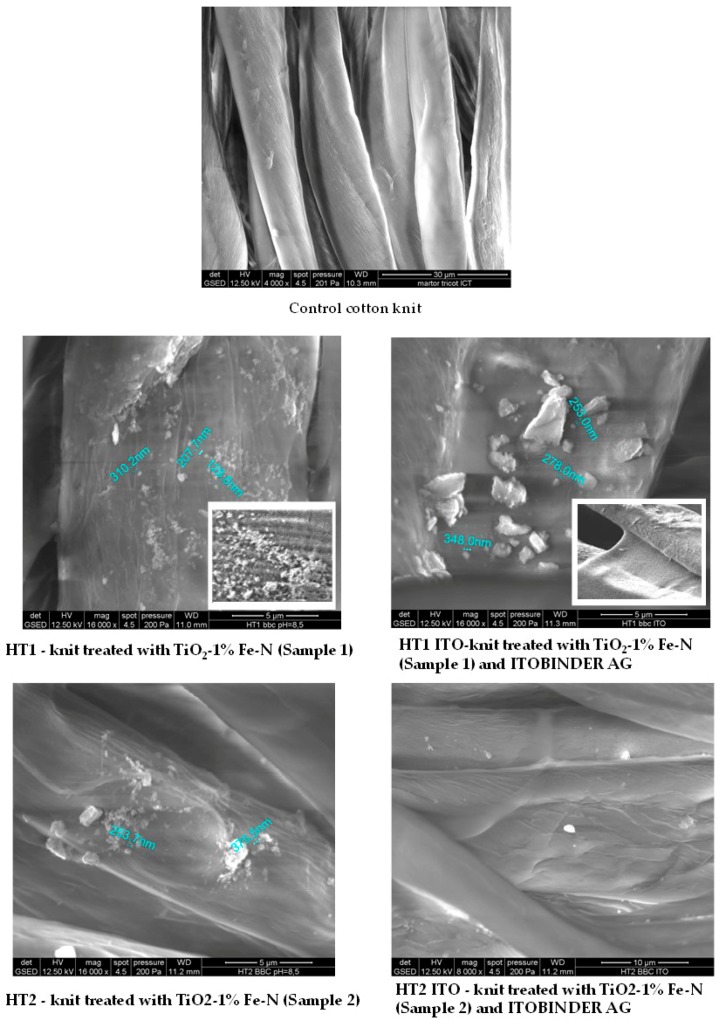
SEM analysis of untreated (control) and treated cotton fabrics.

Scanning electron microscopy analysis (SEM/EDX) of untreated and treated fabrics is presented in Figure 4 and Table 3.

The cotton knit treated with TiO_2_-1% Fe-N Sample 1 (named HT1) was randomly covered with agglomerated particles, whose minimum sizes are 128.8, 207.7, 310.2 nm. The treatment with Sample 2 (HT2—knit treated with TiO_2_-1% Fe-N Sample 2) determined the deposition of a lower number of particles, more agglomerated, with higher dimensions (253.7, 375.5 nm) in comparison with HT1.

On the fibers’ surface treated with Sample 1 and acrylic binder ITOBINDER AG (HT1 ITO), the particles were rare and very large, due to the agglomeration caused by the polyacrylic binder. In the case of HT2 ITO (the knit treated with TiO_2_-1% Fe-N sample 1 and ITOBINDER AG), the TiO_2_-1% Fe-N particles could not be observed properly due to the acrylic polymer that coated the fibers in relatively thick layers. The percentage weights of Ti K are shown in the Table 3.

The amount of photocatalyst deposited on the materials was dependent on the characteristics of the dispersions and the photocatalysts used. Thus, the treatment of textiles by method P2, (represented by the immersion of the textile in the dispersion remained from the first treatment (named method P1) in which ITOBINDER AG was added to better fix the particles on the material’s surface) with liquid that remained from the first treatment with TiO_2_-1% Fe-N (Sample 1) has doubled the amounts of photocatalysts deposited, compared to those deposited by the first treatment (method P1). In the case of TiO_2_-1% Fe-N (Sample 2), the treatment of textiles by method P2 slightly decreased the amounts deposited on cotton fabrics.

### 2.3. Assessment of the Photocatalytic Effects of the Materials Treated with TiO_2_-1% Fe-N Samples

The color differences of the materials treated with photocatalysts, stained with methylene blue and exposed to light irradiation are shown in Table 4 and Table 5. Positive values of the brightness difference (dL*) demonstrated the color lightening of 100% cotton knit exposed to visible light, as compared to unexposed control samples. The most intensive discoloration after staining with MB was observed for the cotton knit treated with the dispersion remaining from the first impregnation with Sample 1 and acrylic binder (HT1 ITO) and on which surface the largest amount of TiO_2_ was deposited. The cotton fabric treated with the dispersion remaining from the first impregnation with Sample 2 and acrylic binder (HT2 ITO) has also shown a severe discoloration. The cotton knit treated with Sample 1 and stained with MB showed almost a triple discoloration (dL* = 5.48) comparing with those treated with Sample 2 (dL* = 2.07). In the case of cotton knit stained with coffee, a very slight discoloration was noticed.

Color difference (dE*) and brightness (dL*) were slightly higher than those of the non-exposed samples. The highest values were recorded on the knits treated with the remaining solution from the first impregnation with Sample 1 and acrylic binder (HT1 ITO) followed by HT1, regardless of the stain type. The polyacrylic binder delays the photodegradation of both coffee and MB. Between the untreated and treated material, there was a difference of 0.5–1 grade on the gray scale, indicating a more rapid degradation of stains due to the applied treatments.

After UV irradiation, the cotton fabric treated with sample 1 (HT1 ITO) and stained with MB showed the most intense discoloration (dL* = 9.41), the largest color difference (dE* = 14.46) and the lowest grade on the scale gray (grade = 1) followed by fabric treated with Sample 2 (HT2 ITO and HT2). The materials stained with coffee showed higher values than the untreated control material demonstrating a reduced photocatalytic activity.

Under natural sunlight, the only material that presented higher brightness values and color differences and lower grades on the gray scale when compared to the untreated cotton knit was the fabric treated with Sample 1 (HT1) and stained with MB (Table 6). It is important to mention that a greater amount of TiO_2_ was deposited on its surface compared to the same material treated with Sample 2. The positive values of da* indicated the red shift of the samples color exposed to solar radiation, being more pronounced in the case of materials treated with photocatalysts. This shift, combined with positive db* values, which indicate the sample yellowing, demonstrate the dye degradation.

Comparing the dL* values after samples’ exposure at UV and visible light (Table 4 and Table 5, respectively), it is obvious that the discoloration was more intense under visible light due to the TiO_2_ doping with nitrogen and iron, which extended the TiO_2_ absorption range. Except sample HT2 ITO, as the TiO_2_ amount deposited on materials increased, so was the photocatalytic efficiency enhanced under visible light.

### 2.4. Antimicrobial Activity

In this study, we report for the first time the antimicrobial activity and biocompatibility of cotton textiles impregnated with TiO_2_-1% Fe-N, which were obtained by using the co-precipitation method at different pH values.

**Figure 5:**
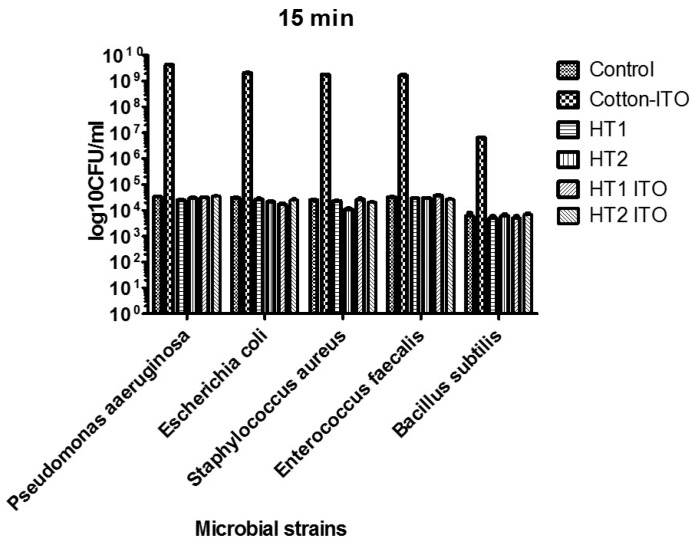
Graphic representation of the log_10_ colony forming units (CFU)/mL values of microbial biofilm cells grown on the cotton knit samples after 15 min of incubation.

The tested materials exhibited specific antimicrobial features, depending on the tested microbial strain and the incubation time. All treated cotton knit materials inhibited the *E. coli* growth after 15 min of incubation. Statistically-significant results as compared to the untreated material have been obtained for HT1 (*p* < 0.01) and HT1 ITO (*p* <0.001). Furthermore, the treated materials, except HT2 ITO, inhibited the *P. aeruginosa* growth, but significant differences compared to the untreated material have been noticed only for HT1 (*p* < 0.01). Excepting HT1 ITO, the treated materials inhibited the growth of the Gram-positive cocci strains, i.e., *S. aureus* and *E. faecalis*, with statistically significant results in the case of HT2 material against *S. aureus* (*p* < 0.001). HT1 and HT1 ITO inhibited the growth of *B. subtilis* after 15 min of incubation (Figure 5). Each of the tested materials inhibited preferentially the growth of a certain microbial strain, i.e., HT1 was the most efficient against *P. aeruginosa*, HT1 ITO against *E. coli* and HT2 against *S. aureus*.

The results were quantified only for three of the five tested bacterial strains, i.e., *P. aeruginosa*, *E. coli* and *S. aureus*, while for *B. subtilis* and *E. faecalis*, no microbial growth was obtained after 24 h of incubation, except for the untreated cotton samples containing only the acrylic binder. 

**Figure 6:**
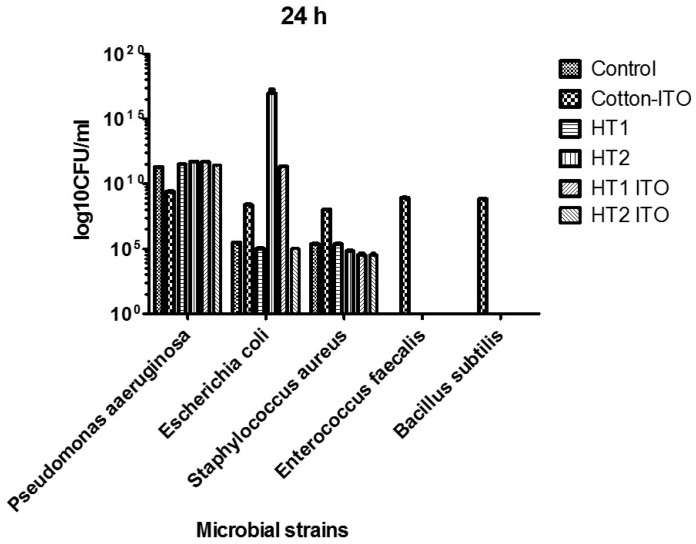
Graphic representation of the logarithmic values of CFU/mL of viable cell counts recovered after 24 h of contact with the cotton knit samples.

For *P. aeruginosa*, the efficiency of the tested materials after 24 h was lower in comparison to that exhibited after 15 min of contact. The number of viable cells obtained in the presence of treated materials was similar to or even higher than that obtained on the control material (Figure 6). These results could be explained by the fact that the particles included in the tested material diffuse rapidly in the culture medium and exhibit a rapid bactericidal effect, decreasing the number of viable cells present in the initial bacterial suspension. However, after the consumption of the antimicrobial agent, the remaining viable *P. aeruginosa* cells start to multiply exponentially, reaching higher densities as compared to those of the initial inocula.

In the case of *E. coli*, the HT1 and HT2 ITO exhibited only a slight inhibitory effect (Figure 6). The HT2 samples, as well as both treated cotton knit containing the ITO acrylic binder, inhibited the growth of *S. aureus* at 24 h. However, the differences registered in the number of viable cells that developed on the treated fabrics were not statistically significant (*p* > 0.05).

These results could suggest that the addition of the polyacrylic binder, despite any intrinsic antimicrobial activity (as revealed by the high microbial load quantified on the untreated cotton samples containing ITO), could increase the antimicrobial activity of the treated materials by prolonging the inhibitory effect (Table 7).

### 2.5. Biocompatibility of Cotton Knit Treated with TiO_2_-1% Fe-N Samples

**Figure 7:**
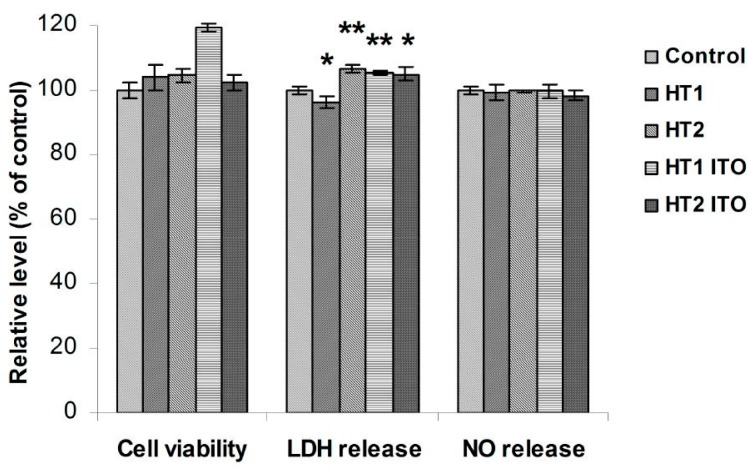
Biocompatibility of cotton knit treated with TiO_2_-1% Fe-N samples after 4 h of incubation with dermal fibroblast cells as shown by cell viability, lactate dehydrogenase (LDH) release and NO level. Results are expressed as the mean ± SD (*n* = 3) and represented relative to the untreated cotton sample (control). * *p* < 0.05 and ** *p* < 0.01 compared to the control.

Taking into account that particles-treated textiles are intended to be marketed due to their self-cleaning and antibacterial properties, the biocompatibility of these materials is essential and should be rigorously assessed in order to check any possible risk of harmful effects that could be induced to the skin, such as cytotoxicity or inflammation. To fulfill this aim, the biocompatibility of TiO_2_-1% Fe-N-treated cotton knit was tested on normal human dermal fibroblasts, which were analyzed for viability, cell membrane integrity and inflammatory status (Figure 7). The period of incubation was selected at 4 h, as this is considered the average time at which the toxic effects are visible on the skin during clothes wearing or after a contact with such functionalized fabrics. 

The cell viability assessed by the 3-(4,5-dimethylthiazol-2-yl)-2,5-diphenyltetrazolium bromide (MTT) test was not significantly modified after exposure to TiO_2_-1% Fe-N, and only the HT1 ITO sample induced a slight increase of the optical density, suggesting that fibroblasts maintained their viability in the presence of particles-treated cotton knit. The level of lactate dehydrogenase (LDH) released in the culture medium during incubation with the treated samples remained near the value measured for the control material. Thus, no damage was induced to the cell membrane integrity after 4 h of direct contact with photocatalyst-treated materials. In addition, these data correlate with the level of NO released in the media, which did not change compared with the control, highlighting the lack of inflammation after 4 h of incubation. According to ISO 10993-5:2009 “Biological evaluation of medical devices-part 5: Tests for in vitro cytotoxicity” [23], these results indicate the biocompatibility of the treated samples meeting the criteria for further in vivo investigation for large-scale production on self-cleaning clothes market. 

**Figure 8:**
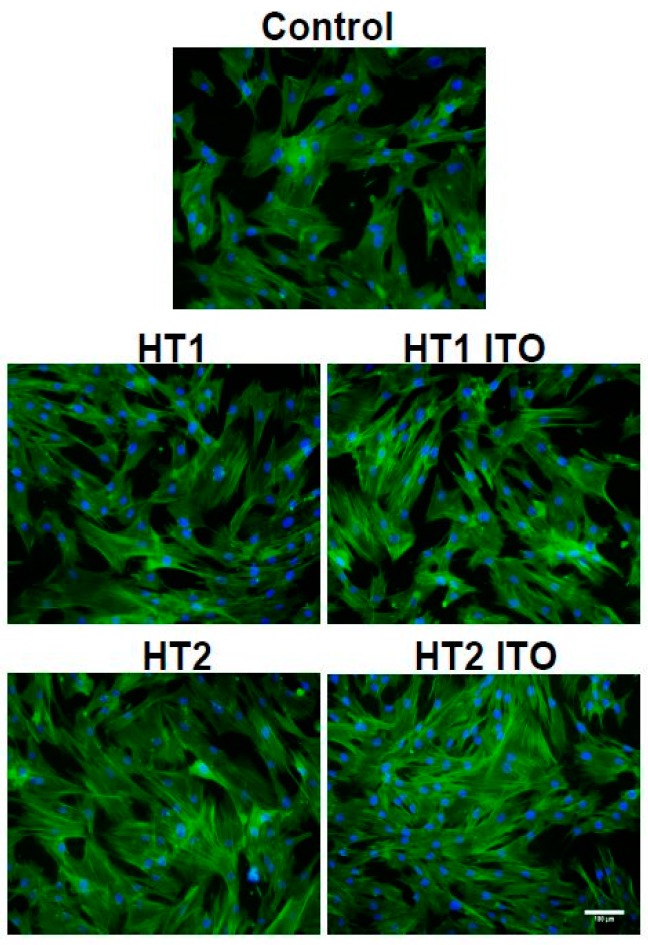
Actin cytoskeleton organization of dermal fibroblast cells after 4 h of incubation with cotton knit treated with TiO_2_-1% Fe-N samples. F-actin (green) was labeled with phalloidin-fluorescein isothiocyanate (FITC), and nuclei (blue) were counterstained with 4′,6-diamidino-2-phenylindole dihydrochloride (DAPI). Scale bar: 100 µm.

In order to assess the cell morphology during incubation with TiO_2_-1% Fe-N-treated fabrics, the organization of the actin cytoskeleton was investigated by fluorescence microscopy (Figure 8). The human dermal fibroblast cell behavior was not significantly influenced in response to the photocatalyst-containing fabrics compared with the control, which is in accordance with the biocompatibility tests shown in Figure 7. The elongated flattened morphology and numerous focal adhesions between cells were maintained after 4 h of incubation with no significant differences between samples.

## 3. Discussion

Iron- and nitrogen-doped nanoscaled titania (TiO_2_-1% Fe-N) was synthesized under hydrothermal conditions at moderate temperature and pressure (200 °C, 15 × 10^5^ Pa). Samples obtained at pH values of 8.5 and 5.5 were further calcined at 400 °C for 2 h. Both samples consisted of a mixture of nanoscaled (8–12 nm) anatase and brookite. Sample 1, synthesized at pH = 8.5, exhibited the best photocatalytic activity under UV and visible irradiation. 

Photocatalysts’ dispersions remaining from the first treatment of textiles contain remarkable quantities of particles that can be re-used by treating the textile materials in the same bath. For better uptake and fixation of these particles, it is necessary to use a binder. The experimental results indicated that by reusing dispersions of TiO_2_-1% Fe-N, a high amount of photocatalysts was deposited on the fabric’s surface, and the photocatalytic activity to decolorize methylene blue dye was improved. Comparing the results of irradiation with UV and visible light, we found that discoloration was more intense under visible light because of the TiO_2_ doping, which extended light absorption in the visible range. Applied on cotton fabrics, Sample 1 showed better photocatalytic efficiency under visible light when compared to Sample 2, while under UV rays; Sample 1 was less active than Sample 2, demonstrating the substrate importance for the photocatalytic activity. 

The treated cotton knits exhibited specific antimicrobial features, depending on their composition, microbial strain and incubation time. The most susceptible microbial strain to the tested materials proved to be *E. coli*, followed by *P. aeruginosa* and the Gram-positive cocci strains. The most significant antimicrobial effect was observed after 15 min of incubation, the most effective materials being HT1 against *P. aeruginosa*, HT2 against *S. aureus* and *E. coli* and HT1 ITO against *E. coli*. Taking into account the implication of these strains in a wide range of infectious diseases, which can occur through direct or indirect contact contamination, the obtained results look promising for the further use of the obtained fabrics in the development of novel textiles with improved antimicrobial properties. In addition, the in vitro biocompatibility evaluation on dermal fibroblast cells confirmed the absence of cytotoxicity after short-term exposure, sustaining the possible use of these innovative cotton textiles after further studies on the long-term skin exposure effects and the possible particle release due to wearing.

## 4. Materials and Methods 

### 4.1. Synthesis of Photocatalysts

Two samples of TiO_2_ doped with 1% Fe atoms and nitrogen were used as photocatalysts, starting from easily-accessible materials (TiCl_3_, FeCl_3_ and urea). For the synthesis of 1 at% Fe-N co-doped TiO_2_, the calculated amounts of FeCl_3_·6H_2_O and TiCl_3_ were introduced under vigorous stirring in distilled water. The pH value was adjusted to 8.5 for Sample 1 and 5.5 for Sample 2 with 25% NH_4_OH solution. The precipitate of Ti (III) hydroxide was oxidized at room temperature with oxygen (air) until the color changes from blue-violet to white. The co-precipitated form of Ti (IV) and Fe (III) hydroxides was washed with distilled water in order to remove salts and then dried in air at 105 °C. For the nitrogen doping, the as-resulted co-precipitate was hydrothermally treated at 200 °C for 2 h in the presence of urea, in a Teflon-lined autoclave. Finally, the dried powder was calcined at 400 °C for 2 h in air.

### 4.2. Preparation of the Photocatalysts for Deposition on Textiles 

Amounts of 0.2 g photocatalyst powder and 0.006 g polyvinylpyrrolidone (PVP), respectively, were introduced in 400 mL of distilled water and stirred for 60 min in an ultrasonic bath. The final pH was adjusted to 8.5 with 1 N NaOH, which is relatively distant from pHpzc (point of zero charge) of TiO_2_, diminishing the repulsion forces between the particles and, consequently, their agglomeration. In addition, NaOH exhibits a swelling effect on textiles, especially on cotton, favoring the absorption of the photocatalysts.

PVP was used in formulating photocatalysts’ dispersions due to its ability to form molecular thin films on the surface of the particles, thereby preventing their agglomeration and sedimentation. Furthermore, PVP is a good moisturizer, absorbing over 40% of atmospheric moisture and, thus, contributing to the improvement of the photocatalytic effects. 

### 4.3. Cotton Knit Treatment 

#### 4.3.1. Method P1

One hundred percent cotton knit (213 g/m^2^, 148/137.5 wales/courses; 1.08 mm thickness) was washed at 90 °C with water to remove any contaminants and detergent traces. The washed material was immersed in the above prepared photocatalyst dispersions, sonicated for 60 min at 40 °C and then dried in an oven at 100 °C for 60 min. It is important to note that a large amount of particles remained in the dispersion after this first treatment.

#### 4.3.2. Method P2

In order to increase the degree of exhaustion of the treatment bath and to reduce the amount of TiO_2_ particles introduced into wastewaters, new textile materials were immersed in the remaining dispersion and treated in similar conditions as above. To fix the particles, the wet materials were immersed in 20 mL/L polyacrylic binder (ITOBINDER AG, self-cross linking aqueous acrylic copolymer emulsion acquired from LJ Specialities, Holmewood, UK, abbreviated ITO), maintained in an ultrasound bath for 30 min and then dried at 100 °C.

For comparisons, the fabric was treated with 400 mL solution containing 0.006 g PVP and 20 mL/L ITO.

The treated materials were noted: cotton ITO, knit treated with PVP and ITO; HT1, knit treated with TiO_2_-1% Fe-N (Sample 1); HT2, knit treated with TiO_2_-1% Fe-N (Sample 2); HT1 ITO, knit treated with TiO_2_-1% Fe-N (Sample 1) and ITO; HT2 ITO, knit treated with TiO_2_-1% Fe-N (Sample 2) and ITO. 

### 4.4. Photocatalyst Characterization

The crystallization of photocatalysts obtained under hydrothermal conditions followed by thermal annealing at 400 °C for 2 h was highlighted by X-ray diffraction (XRD). A Bruker D8 Advance diffractometer (Bruker, Hamburg, Germany) using Cu Kα radiation (λ = 1.5406 Å) was used. The presence of iron in the TiO_2_ lattice was revealed by room temperature ^57^Fe Mössbauer transmission spectra recorded using a WissEL-ICE Oxford Mössbauer cryomagnetic system (Wissenschaftliche Elektronik GmbH, Starnberg, Germany, and ICE Innovative cryogenic system, Oxford, UK). The photocatalytic properties of Samples 1 and 2 (films on quartz) in the degradation of methylene blue, in both UV and visible regions, were investigated by means of the PCC2- ULVAC photocatalytic checker (ULVAC, Chigasaki, Kanagawa, Japan). 

The morphology of the fabrics’ surfaces treated with photocatalysts and the amount of deposited metals were analyzed by SEM (Quanta 200, FEI, Eindhoven, The Netherlands) equipped with an EDX detector. Assessment of the photocatalytic effects was performed by staining the blank materials and those treated with TiO_2_-1% Fe-N (Sample 1 and Sample 2) with 0.01 g/L MB solution and coffee (3 coffee teaspoons in 500 mL water). The materials were half covered with paper and were exposed to UV light (254 nm) in a completely closed cabinet and to visible light in a laboratory equipment, Xenotest. Chromaticity coordinates of exposed and non-exposed materials were measured by an UltraScan PRO Hunterlab UV-Vis spectrophotometer (Hunter Associates Laboratory, Reston, VA, USA). The color differences between the treated samples and the blank were measured via the CIE L*a*b* scale specified by the “Commission Internationale de l’Éclairage” (CIE) [23], where:

- L* represents lightness, a* signifies the red/green value and b* is the yellow/blue value;
- dL* = L*sample − L*control; if dL* is positive, the sample is lighter than the control and, if dL* is negative, the sample is darker than the control;
- da* = a*sample − a* control; if da* is positive, the sample is redder than the control. If da* is negative, the sample is greener than the control;
- db* = b*sample − b* control; if db* is positive, the sample is yellower than the control. If db* is negative, the sample is bluer than the control;
- dE* represents the total difference or distance on the CIELAB diagram as a single value for colour (da*, db*) and lightness (dL*) and, is calculated according to the following formula: dE* = $\sqrt{dL{*}^{2}+da{*}^{2}+db{*}^{2}}$.

### 4.5. Antimicrobial Activity Assay

The Gram-positive *S. aureus* ATCC 6538, *E. faecalis* ATCC 29212 and *B. subtilis* ATCC 6633 and the Gram-negative *P. aeruginosa* ATCC 27853 and *E. coli* ATCC 8739 were purchased from American Type Culture Collection (ATCC, Virginia, USA). Glycerol stocks were streaked on LB agar to obtain 24-h cultures to be used for all further studies.

Monospecific biofilm development was assessed at two different times of exposure, i.e., 15 min and 24 h. The textile materials were cut in equal circular samples of 8 mm and sterilized by autoclaving at 121 °C for 15 min. The sterile samples were then immersed in 1 mL of microbial suspensions of ~10^7^ colony forming units (CFU)/mL performed in sterile saline and left in contact for 15 min and 24 h, respectively. After this interval, microbial suspensions incubated with the tested samples were vortexed and further serially ten-fold diluted, and 10 µL of each serial dilution were plated in triplicate on LB agar. After 24 h of incubation at 37 °C, viable cell counts were performed, and the number of CFU/mL for each sample was established.

### 4.6. In Vitro Biocompatibility Assessment

CCD-1070Sk normal human skin fibroblasts (purchased from ATCC, Cat. No. CRL-2091) were cultured at low passage in complete Eagle’s minimum essential medium (MEM; Gibco/Invitrogen, Carlsbad, CA, USA) containing 10% fetal bovine serum (FBS; Gibco/Invitrogen) at 37 °C in a humidified atmosphere with 5% CO_2_. Fibroblasts were grown to 70%–80% confluence within five to six days, then detached with 0.25% trypsin-0.03% EDTA and transferred to new culture flasks. The cells were seeded at a density of 2 × 10^4^ cells per square centimeter in a 24-well plate and were left to adhere overnight. Then, cotton samples cut into 1 cm × 1 cm, sterilized at 120 °C for 20 min and exposed to a visible light source for 30 min, were soaked in culture medium and placed over the attached fibroblasts without disturbing the cells, as was previously reported for other materials [24]. After 4 h of incubation, cytotoxicity tests were performed, and cell morphology and viability were evaluated. The untreated cotton sample (control) was used a control, and the results were expressed relative to this one.

The cell viability was measured using the 3-(4,5-dimethylthiazol-2-yl)-2,5-diphenyltetrazolium bromide (MTT; Sigma-Aldrich, St. Louis, MO, USA) assay (from the battery of cytotoxicity tests described in ISO 10993-5:2009, part 5) [25] which is based on the quantification of mitochondrial succinate dehydrogenase activity in the viable cells. Briefly, the culture medium and cotton samples were removed at the end of the exposure time, and cells were incubated with 1 mg/mL MTT for 3 h at 37 °C and 5% CO_2_. The purple formazan crystals formed in the viable cells were dissolved with 2-propanol (Sigma-Aldrich, St. Louis, MO, USA), and the absorbance was measured at 595 nm using a microplate reader (GENiosTecan, Salzburg, Austria).

The LDH release was assessed as a measure of cell membrane integrity using a commercial kit (TOX7, Sigma-Aldrich, St. Louis, MO, USA) according to the manufacturer’s instructions. Volumes of 50 µL of culture supernatants were incubated with 100 µL mix composed of equal parts of dye, substrate and cofactor for 30 min. The reaction was stopped by adding 15 µL of 1 N HCl, and the absorbance was read at 490 nm using a GENiosTecan microplate reader. 

The level of nitric oxide (NO) released in the culture medium as an indicator of inflammation was determined using the Griess reagent. Culture supernatants were mixed with an equal volume of Griess reagent, a stoichiometric solution (v/v) of 0.1% naphthylethylenediamine dihydrochloride and 1% sulfanilamide in 5% H_3_PO_4_. Absorbance was read at 550 nm using a GENiosTecan microplate reader, and the NO concentration was calculated on a NaNO_2_ standard curve.

Cell spreading and actin cytoskeleton morphology were investigated by fluorescence imaging using cells fixed with 4% paraformaldehyde for 20 min and permeabilized with 0.1% Triton X-100-2% bovine serum albumin for 1 h. Images were captured using an inverse fluorescence microscope Olympus IX71 (Olympus, Tokyo, Japan). Filamentous actin (F-actin) was labelled with 20 µg/mL phalloidin conjugated with fluorescein isothiocyanate (FITC) (Sigma-Aldrich, Munich, Germany), and nuclei were stained with 2 µg/mL 4′,6-diamidino-2-phenylindole dihydrochloride (DAPI) (Sigma-Aldrich, Munich, Germany).

### 4.7. Statistical Analysis

The antimicrobial activity and cell culture assays were performed in triplicate, and data were presented as the mean ± standard deviation (SD). The statistical significance was analyzed by Student’s *t*-test or two-way analysis of variance (ANOVA) followed by the Bonferroni *post hoc* test using GraphPad Prism 5, and a value of *p* < 0.05 was considered significant.

## Figures and Tables

**Table 1:** Rietveld refinement parameters for TiO_2_-1% Fe-N hydrothermal samples.

Sample	Lattice Parameters (Å)			Crystallite Size (nm)	Phase Assignment/Abundance (wt %)
	*a*	*b*	*c*		
Sample 1 (pH = 8.5)	3.7912	-	9.4909	12.3	Anatase 85.3
	9.1429	5.4215	5.2450	8.5	Brookite 14.7
Sample 2 (pH = 5.5)	3.7907	-	9.4743	10.4	Anatase 79.4
	9.1629	5.4437	5.1809	11.6	Brookite 20.6
Errors	±0.0005	±0.0005	±0.0005	±1.5	±1.4

**Table 2:** Mössbauer fit parameters for the for TiO_2_-1% Fe-N hydrothermal samples. IS, isomer shift.

Sample	IS (mm/s)	ΔE_Q_ (mm/s)	Γ (mm/s)	Areas (%)	Site/Phase Assignment
Sample 1	0.417	0.598	0.45	85.2	Fe^3+^: Anatase
	0.096	0.703	0.46	14.8	Fe^3+^: Brookite
Sample 2	0.419	0.551	0.40	79.4	Fe^3+^: Anatase
	0.182	0.587	0.41	20.6	Fe^3+^: Brookite
Errors	±0.002	±0.004	±0.03	±0.4	-

**Table 3:** EDX analyses of the cotton knit treated with TiO_2_-1% Fe-N (Samples 1 and 2) and polyacrylic binder.

Element, wt %	HT1	HT1 ITO	HT2	HT2 ITO
C K	46.19	45.99	49.20	68.01
O K	43.71	34.93	45.77	29.44
**Ti K**	**10.09**	**19.07**	**5.04**	**2.55**
Total	100	100	100	100

**Table 4:** Trichromatic coordinates of the cotton knit treated with TiO_2_-1% Fe-N (Samples 1 and 2), stained with 0.01 g/L methylene blue (MB) and coffee exposed to visible light (Xenotest).

Sample	L*	a*	b*	dL*	da*	db*	dE*	dC*	dH*	Grades
Control cotton knit stained with MB	86.98	−2.06	−2.53	0.23	5.62	5.14	7.62	−7.59	0.61	2
Cotton knit ITO-MB	82.32	−3.83	−7.02	2.46	1.04	1.23	2.94	−1.58	0.30	3.50
HT1-MB	84.75	−4.32	−2.93	5.48	13.37	9.68	17.39	−16.5	−0.25	1
HT1 ITO-MB	90.35	−2.01	−0.34	13.31	14.66	13.27	23.84	−19.48	−3.39	1
HT2-MB	82.13	−6.05	−5.68	2.07	11.82	7.59	14.2	−13.96	1.57	1
HT2 ITO-MB	89.68	−3.07	−1.47	5.92	14.92	10.49	2.2	−18.2	−1.2	1
Control cotton knit stained with coffee	80.26	4.96	19.72	3.46	−0.68	0.45	3.56	0.26	0.77	3
Cotton knit ITO-coffee	71.95	6.86	22.17	1.82	−0.72	−2.42	3.12	−2.53	−0.03	3.50
HT1-coffee	78.38	5.2	21.92	5.06	−0.91	0.6	5.18	22.18	-	2.5
HT1 ITO-coffee	82.25	4.99	22.61	4.88	−1.33	0.68	5.1	0.34	1.46	2.5
HT2-coffee	81.29	5.25	23.69	4.05	−1.16	0.6	4.25	0.3	1.27	2.5
HT2 ITO-coffee	80.98	5.56	25.26	2.9	−0.67	1.54	3.35	1.34	1.01	3

**Table 5:** Trichromatic coordinates of the cotton knit treated with TiO_2_-1% Fe-N (Samples 1 and 2), stained with 0.01 g/L MB and coffee, exposed to UV light.

Sample	L*	a*	b*	dL*	da*	db*	dE*	dC*	dH*	Grades
Control cotton knit stained with MB	87.38	−5.06	−2.61	3.2	5.68	6.58	9.26	−8.44	−2.07	1.5
Cotton knit ITO-MB	77.67	−18.76	−15.15	−2.36	−0.61	−0.19	2.44	0.59	−0.24	3.50
HT1-MB	81.7	−8.01	−3.02	1.7	7.09	8.03	10.84	−10.15	−3.42	1.5
HT1 ITO-MB	−8.47	−1.55	2.12	9.41	11.52	15.03	14.46	−13.54	−6.17	1
HT2-MB	82.13	−6.05	−5.68	2.07	11.82	7.59	14.2	−13.96	1.57	1
HT2 ITO-MB	87.76	−7.04	2.31	2.93	7.46	10.02	12.83	−9.02	−8.65	1
Control cotton knit stained with coffee	73.19	5.96	21.56	−5.72	1.71	5.22	7.93	5.49	−0.29	2
Cotton knit ITO-coffee	67.97	7.91	27.01	1.41	−0.68	−1.58	2.22	−1.71	0.20	4.00
HT1-coffee	74.76	5.13	21.55	0.4	−0.01	2.18	2.22	2.11	0.55	4
HT1 ITO-coffee	75.47	7.31	24.87	3.06	−1.12	−0.30	3.27	−0.63	0.98	3
HT2-coffee	76.05	5.28	24.39	1.97	−0.45	3.18	3.77	2.99	1.18	3
HT2 ITO-coffee	74.83	6.01	24.71	2.07	−0.77	2.26	2.97	1.98	1.34	3.5

**Table 6:** Trichromatic coordinates of the cotton knit treated with TiO_2_-1% Fe-N (Samples 1 and 2), stained with 0.01 g/L MB and coffee and exposed to solar light.

Sample	L*	a*	b*	dL*	da*	db*	dE*	dC*	dH*	Grades
Control cotton knit stained with MB	85.7	−3.41	−4.67	4.85	5.59	4.58	8.71	−7.13	1.21	1.5
Cotton knit ITO-MB	78.79	−10.54	−11.62	0.08	9.36	4.93	10.58	−10.20	2.83	1.50
HT1-MB	88.5	−1.68	−2.87	5.93	11.48	7.19	14.79	−13.24	2.86	1
HT2-MB	85.36	−3.02	−2.4	1.58	7.77	6.53	10.27	−10.15	−0.14	1.5
Control cotton knit stained with coffee	69.48	8.07	26.77	0.23	−1.08	−3.57	−3.73	0.01	1.01	3.5
Cotton knit ITO-coffee	68.44	7.21	24.32	−1.43	−0.64	−3.35	3.70	−3.40	−0.32	3.00
HT1-coffee	78.17	4.64	21.04	2.54	−0.59	1.25	2.9	1.08	0.87	3.5
HT2-coffee	80.26	3.71	17.58	5.8	−2.16	−3.38	7.05	−3.8	1.29	2

**Table 7:** Representation of the material samples exhibiting antimicrobial activity against the tested microbial strains at different time intervals.

Microbial Strain		15 min	24 h
Gram negative bacilli	*P. aeruginosa*	HT1, HT2, HT1 ITO	-
	*E. coli*	HT1, HT2, HT1 ITO, HT2 ITO	HT1, HT2 ITO
Gram positive bacilli	*B. subtilis*	HT1, HT1 ITO	-
Gram positive cocci	*S. aureus*	HT1, HT2, HT2 ITO	HT2, HT1 ITO, HT2 ITO
	*E. faecium*	HT1, HT2, HT1 ITO	-
